# Microsurgical Clipping of Unruptured Middle Cerebral Artery Bifurcation Aneurysms: A Single-Center Experience

**DOI:** 10.3390/brainsci14111068

**Published:** 2024-10-26

**Authors:** Nico Stroh-Holly, Philip Rauch, Harald Stefanits, Philipp Hermann, Helga Wagner, Michael Sonnberger, Maria Gollwitzer, Stefan Aspalter, Andreas Gruber, Matthias Gmeiner

**Affiliations:** 1 Department of Neurosurgery, Kepler University Hospital, Johannes Kepler University Linz, Wagner-Jauregg-Weg 15, A-4020 Linz, Austria; 2 Center for Clinical Studies (CCS Linz), Johannes Kepler University Linz, A-4040 Linz, Austria; 3 Institute of Applied Statistics, Johannes Kepler University Linz, A-4040 Linz, Austria; 4 Institute of Neuroradiology, Kepler University Hospital, Johannes Kepler University Linz, A-4040 Linz, Austria; 5 Clinical Research Institute for Neuroscience, Johannes Kepler University Linz, A-4040 Linz, Austria

**Keywords:** intracranial aneurysm, unruptured intracranial aneurysm, middle cerebral artery aneurysm, clipping, microsurgical treatment, outcome

## Abstract

Background/Objectives: Microsurgical clipping has traditionally been considered a standard treatment for middle cerebral artery (MCA) aneurysms. Recently, a caseload reduction related to improved endovascular treatment options has occurred in cerebrovascular neurosurgery. Therefore, studies that report the clinical and radiological outcomes after clipping are highly warranted. Methods: Patients with an unruptured MCA bifurcation aneurysm, who were surgically treated at the Department of Neurosurgery in Linz between 2002 and 2019, were included in this study. Clinical and radiological outcome parameters were evaluated for each patient. Results: Overall, 272 patients were eligible for inclusion. Complete aneurysm occlusion was demonstrated in 266 (99.3%) of the 268 (98.5%) patients who underwent postoperative digital subtraction angiography. In six (2.2%) patients, a permanent new neurological deficit (pNND) persisted after treatment. Intraoperative aneurysm rupture was a significant factor (*p* = 0.0049) in the logistic regression. At the last follow-up, only two patients (0.7%) had an unfavorable outcome (mRS > 2). More recent surgeries were associated with fewer cases of pNND (*p* = 0.009). A transient new neurological deficit occurred in 13 patients (4.8%), with aneurysm size being a significant risk factor (*p* = 0.009). Surgical site infections were reported in four patients (1.5%), with patient age (*p* = 0.039) and time (*p* = 0.001) being significant factors. Two patients died (0.7%) perioperatively and two patients (0.7%) needed a retreatment in the long-term follow-up. Conclusions: The findings indicate that microsurgical clipping is a safe procedure with minimal need for retreatment. It achieves a high occlusion rate while maintaining a very low rate of adverse outcomes. Continuous intraoperative enhancements over time have contributed to a progressive improvement in clinical outcomes in recent years. This trend is exemplified by the absence of detectable pNND in the era of ICG angiography. Consequently, these data support the conclusion that microsurgical clipping should still be considered an appropriate treatment option for unruptured MCA bifurcation aneurysms.

## 1. Introduction

The prevalence of unruptured intracranial aneurysms (UIAs) is estimated at 3%, and their detection has significantly improved, attributable to advancements in and increased accessibility of intracranial imaging techniques [1,2,3]. The natural course of UIAs remains inherently uncertain, and approximations of the rupture risk assessment are available in previous studies [4,5,6]. The risk of aneurysm rupture and consequently of a subarachnoid hemorrhage (SAH) needs to be balanced with the risk of prophylactic aneurysm treatment, regardless of the treatment modality. For predicting clinical and functional outcomes after microsurgical treatment of UIAs, Machine Learning (ML)-based models have been published recently [7]. A multidisciplinary consensus on which aneurysms have a higher rupture risk and therefore which ones need to be treated has already been discussed in several consensus papers [8,9]. The question of the most appropriate treatment method becomes more debatable due to the increasing number of endovascular options [10,11].

Approximately one third of UIAs are middle cerebral artery (MCA) aneurysms [5]. Microsurgical clipping has long been the exclusive treatment strategy for unruptured intracranial aneurysms (UIAs), particularly for those located in the MCA bifurcation [12]. Rapidly emerging modern endovascular therapy approaches, such as coiling, stent- or balloon-assisted coiling, Flow Diverter (FD) or WovenEndoBridge (WEB) Implantation, now aim to compete with the results of the gold standard of microsurgical clipping [13,14,15,16,17,18].

The discourse surrounding the optimal future treatment method for unruptured intracranial aneurysms is dynamic and ongoing. Recent cohort studies highlight the potential of endovascular techniques, pointing to their enhanced long-term occlusion rates and minimally invasive nature as reasons for their increasing adoption [18,19,20].

In terms of occlusion rate, permanent repair, or thromboembolic events, a number of cohort studies still show superior results with microsurgical clipping for unruptured MCA aneurysms [10,21,22,23,24,25]. The anatomical characteristics of unruptured MCA aneurysms, including their wide-necked bases, trifurcated anatomy, and involvement of M2 branches, have traditionally been viewed as challenges for endovascular treatment, making microsurgical clipping a preferred approach for these specific situations [22].

In the context of evolving therapeutic techniques, the need for a detailed evaluation of outcomes and complications across a comprehensive cohort becomes paramount. This retrospective explorative study, by focusing on the microsurgical treatment of 272 unruptured MCA bifurcation aneurysms at a high-volume center over an extended duration of 18 years, aims to contribute valuable insights to the scientific discourse on the most effective treatment modalities for these aneurysms, enhancing our understanding of patient outcomes in the long term.

## 2. Material and Methods

### 2.1. Overview

The retrospective explorative study obtained ethical approval from the local Ethics Committee of the Federal State Upper Austria (EK-No.: 1255/2019). This investigation centered on patients with an unruptured MCA bifurcation aneurysm who received microsurgical clipping treatment at the Department of Neurosurgery, Kepler University Hospital Linz, from January 2002 to October 2019. The patient cohort was derived from our hospital’s surgical database, which is maintained for clinical purposes and quality assurance. Patient data were systematically collected and collated in a retrospective database for subsequent analysis.

### 2.2. Patient-Specific Parameters

Fundamental patient demographics, encompassing age and gender, were extracted from the medical records of the Department of Neurosurgery at the Kepler University Hospital Linz. Additionally, a comprehensive assessment of individual medical histories was carried out, which included an investigation into any previous occurrences of SAH and the confirmed diagnosis of autosomal dominant polycystic kidney disease (ADPKD).

### 2.3. Aneurysm-Specific Parameters

Angiographic imaging was systematically conducted for all patients as a preoperative measure, utilizing either digital subtraction angiography (DSA) or computed tomography angiography (CTA). A meticulous evaluation of various aneurysm-related parameters was undertaken based on the radiographic reports. In the case of missing reports, experienced neuroradiologists conducted a thorough reassessment of the radiographic images.

The size of each aneurysm was stratified into three categories: small (<10 mm), large (10–25 mm), and giant aneurysms (>25 mm) [26]. Additionally, the study evaluated various factors, including the aneurysm’s specific location, the presence of blebs, evidence of calcification or thrombosis, any previous attempts at coiling, and the detection of concurrent aneurysms. The presence of coincident aneurysms was not considered as an exclusion criterion. Only MCA bifurcation aneurysms were included in this study. Thus, aneurysms of the M1 segment, as well as peripheral MCA aneurysms of the M3 and M4 segments, were excluded.

### 2.4. Intraoperative Parameters

The microsurgical procedures were executed by experienced senior neurosurgeons. Intraoperative metrics, including the quantity of clips used, the utilization of intraoperative neuromonitoring through somatosensory evoked potentials (SSEP) and motor evoked potentials (MEP), the application of intraoperative indocyanine green (ICG) angiography, instances of intraoperative aneurysm rupture, and the need for clip repositioning or temporary clipping for proximal vascular control, were systematically extracted from the surgical reports. The surgical technique employed was uniform across all cases, ensuring a standardized and comparable basis for analysis.

### 2.5. Radiological Outcome Parameters

**Figure 1:**
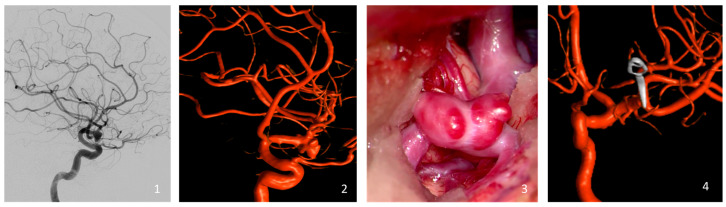
Illustration of a representative case of the cohort (clipping of a left-sided MCA bifurcation aneurysm). Preoperative Digital Subtraction Angiography (**1**) and the reconstructed 3D Rotational Angiography (**2**). Intraoperative view of the MCA aneurysm with its blebs just before the clipping (**3**), and the intraoperative reconstructed 3D Rotational Angiography (**4**).

Aneurysm occlusion was rigorously assessed through either intraoperative or postoperative DSA (Figure 1). Occlusion levels were determined using the Raymond Roy Occlusion Classification (RROC), which organizes occlusion results into three specific categories: Class I, which represents complete occlusion without any remaining aneurysm; Class II, which denotes the presence of a residual neck; and Class III, which indicates the continued existence of a residual aneurysm [27].

This categorization framework provided a nuanced evaluation of the effectiveness of the surgical intervention and was pivotal for discerning the extent of aneurysm closure in a standardized manner.

### 2.6. Clinical Outcome Parameters

To assess morbidity and mortality, an array of postoperative parameters was extracted from the medical records in accordance with the current literature [21,27,28]. These parameters included postoperative intracerebral hemorrhage (ICH), incidents of postoperative epileptic seizures, and occurrences of postoperative surgical site infection (SSI).

New postoperative neurological deficits (NND) were categorized into transient (tNND) and permanent (pNND) categories—with pNND persisting beyond hospital discharge. Additional outcome parameters included chronic subdural hematoma (cSDH), pulmonary embolism (PE), and perioperative mortality.

Reoperations necessitated by the primary surgical procedure were systematically documented as critical outcome variables. Moreover, longitudinal monitoring was employed to assess aneurysm recurrence and the incidence of subarachnoid hemorrhage (SAH) during the follow-up. These measures facilitated a nuanced understanding of postoperative and long-term patient outcomes, crucial for evaluating the procedure’s efficacy and safety.

### 2.7. Statistical Analysis

The statistical analysis was conducted at the Center for Clinical Studies (CCS) of the Johannes Kepler University in Linz. Statistical analysis included descriptive statistics for all valid observations. For nominal variables, absolute and relative frequencies, and for metric variables, mean and standard deviation (SD) as well as median and range were computed. To analyze the association between two nominal variables, Fisher’s exact test was used. Logistic regression models were fitted to model the effects of patient-specific, aneurysm-specific, and intraoperative parameters on the probability of the following outcomes: NND, pNND, tNND, postoperative epileptic seizures, and SSI. For each of these outcomes, a model with all potential regressors not leading to separation was first fitted. Then, stepwise variable selection using the Akaike Information Criterion (AIC) was performed. In all regression analyses, observations with missing values in any of the relevant variables were excluded. All statistical analyses were performed using the statistical software R (R Foundation for Statistical Computing, Version 4.4.1, Vienna, Austria).

## 3. Results

In the analysis, 272 aneurysms were evaluated in total. The patient cohort had a mean age of 55 years, with 76 (27.9%) males and 196 (72.1%) females. Additionally, 42 (15.4%) patients presented with a documented history of SAH, although the origin of the bleeding was not linked to the MCA bifurcation aneurysms being studied. All patient-specific preoperative data are succinctly summarized in Table 1.

Analysis of aneurysm-specific parameters revealed that 151 (55.5%) of the aneurysms were located in the right MCA bifurcation, whereas 121 (44.5%) were found in the left MCA bifurcation. The majority of these aneurysms, accounting for 242 (89%) cases, exhibited a maximum diameter size of the aneurysm smaller than 10 mm, whereas 29 (10.7%) aneurysms measured between 10 and 25 mm. Remarkably, only one (0.4%) aneurysm characterized as giant—with a diameter exceeding 25 mm—was documented within the registry. The calculated average diameter across all aneurysms was 5.8 mm (range 2–25 mm).

Notably, in seven (2.6%) instances, aneurysms were initially treated with coiling and then necessitated subsequent microsurgical intervention due to recurrence. Additionally, preoperative angiography revealed the presence of a daughter aneurysm, resembling a bleb, in 53 (19.5%) cases. It is important to highlight that preoperative DSA was accessible for 241 (88.6%) patients, whereas 31 patients (11.4%) underwent preoperative assessment using CTA. Comprehensive details of the aneurysm-specific preoperative parameters are also summarized in Table 1.

### 3.1. Intraoperative Parameters

The intraoperative parameters are detailed in Table 2. The average number of clips used for aneurysm treatment was 1.5, ranging from 1 to 8. Notably, the repositioning of clips became necessary in 60 (22.4%) cases, and 8 (3.0%) aneurysms experienced rupture during the surgical approach. Temporary clipping of the parenteral vessel was required in 38 (14.1%) instances. Intraoperative monitoring, encompassing MEP and SSEP, was available in 68 (25%) procedures, while intraoperative ICG angiography was used in 102 (37.5%) cases.

### 3.2. Outcome Parameters

In 268 (98.5%) patients, either intraoperative or postoperative DSA was conducted. In 99.3% of cases, the DSA demonstrated a RROC Class I occlusion—a complete occlusion without any evidence of a residual neck or aneurysmal remnants. Three patients (1.1%) were identified with an ICH necessitating surgical revision. These hemorrhages were associated with the surgical approach, and aneurysm-related secondary bleeding was conclusively ruled out in all instances.

Postoperatively, NND was observed in 19 (7%) patients, with 6 (2.2%) patients exhibiting pNND and 13 (4.8%) patients experiencing tNND. Eleven (4%) patients presented with epileptic seizures for the first time postoperatively. SSI necessitating revision occurred in four (1.7%) cases, and cSDH requiring burr hole trepanation at follow-up was observed in three (1.1%) cases. Two patients died postoperatively, and therefore overall mortality was 0.7% (fulminant sepsis, pulmonary embolism). Additionally, two (0.7%) patients experienced recurrent aneurysms in the subsequent follow-up—both underwent surgical retreatment. Details of all postoperative radiological and clinical parameters are presented in Table 3 and Table 4.

### 3.3. Logistic Regression for Outcome Parameters

Logistic regression analyses were conducted for the outcome parameters (NND, pNND, tNND, epileptic seizures postoperative and SSI). For all these models, at least 265 (97.43%) valid observations without any missing values were used for model fitting. Table 4, Table 5, Table 6, Table 7 and Table 8 present the results from the full logistic regression models (with all covariates except those leading to separation) and after stepwise variable selection using the AIC. The key findings of the logistic regression models after stepwise variable selection are described here.

The size of the aneurysm exhibited an association with postoperative NND (*p* = 0.001), particularly with tNND (*p* = 0.009). Intraoperative temporary clipping was associated with the occurrence of postoperative epileptic seizures (*p* = 0.037). The occurrence of intraoperative aneurysm rupture is associated with the development of pNND (*p* = 0.049). Risk of pNND is lower in more recent surgeries (*p* = 0.009), but higher with interoperative neuromonitoring (*p* = 0.023). For the association between ICG angiography and pNND, Fisher’s exact test yields a *p*-value of *p* = 0.087.

## 4. Discussion

In this study, we report on a cohort of 272 unruptured MCA bifurcation aneurysms treated with microsurgical techniques, marking it as one of the largest datasets from a single institution examined to date. This study reports a high (99,3%) aneurysm occlusion rate and a low unfavorable outcome rate concerning pNND. Furthermore, the results show a low retreatment rate of 0.7% after microsurgical clipping of unruptured MCA bifurcation aneurysms.

Compared to other series of unruptured MCA bifurcation aneurysms, Nussbaum et al. published a large monocentric, single-surgeon experience, including 716 patients [21]. Further large monocentric case series of microsurgically treated patients with unruptured MCA aneurysms have been presented by Rodriguez et al. (n = 261), Metayer et al. (n = 158), and Morgan et al. (n = 263) [22,28,29].

Our angiographic assessment revealed an aneurysm occlusion rate of 99.3%, aligning closely with the range of 92% to 98.9% reported in the existing literature [11,21,22,23,24,30,31,32]. It is well known from prior benchmark studies that microsurgically treated MCA aneurysms generally exhibit extremely low recurrence rates and very high occlusion rates in angiographic long-term follow-ups. Spetzler et al. demonstrated a long-term occlusion rate of 93% in angiographic controls after 10 years, while Mooney et al. reported an occlusion rate of 95% after 6 years with a mean angiographic follow-up of 3.7 years [33,34]. Rodriguez et al. even reported a long-term recurrence rate of 0% with a mean angiographic follow-up of 3.9 years [22].

Recently published data reveals that complete occlusion rates for MCA aneurysms treated with coiling, with or without adjunctive stent- or balloon-assistance, stands at 29.4% [35]. Further, Bracard et al.’s subanalysis on coiling of unruptured MCA aneurysms indicates a complete occlusion rate of 31.6% [13]. With regard to emerging endovascular approaches, the literature cites initial occlusion rates for WEB devices from 17.7% to 53.8%, with an increase of up to 59.4% observed after a 3-year follow-up [15,19,20]. Meanwhile, for flow diverters (FD), initial occlusion rates are documented at 5–10%, with a subsequent rise to 55.6–82.1% after a period exceeding 12 months [17,18,36,37]. A recent meta-analysis by Tocacelli et al. reported an average occlusion rate of 31.5% in the initial phase and 60% in the long term (beyond 12 months) for all endovascular treatment modalities [10].

The findings of this study validate the conclusions of prior research, indicating that microsurgical intervention for unruptured aneurysms tends to provide superior rates of closure, both in the initial phase and throughout the follow-up period, when contrasted with endovascular treatment modalities [11,24,25,38,39].

The outcomes reveal an exceptionally low retreatment rate, observed in only two (0.7%) patients. Both cases demonstrated complete occlusion in their initial postoperative angiographic evaluations. Recurrence was observed in one aneurysm through routine follow-up imaging, necessitating surgical intervention for correction. The second recurrence led to a more severe outcome, presenting as a ruptured aneurysm resulting in SAH, which also required surgical retreatment. The low retreatment rate observed in this study aligns with the existing literature [21,22,29,30,40]. In contrast, endovascular alternatives show device-dependent retreatment rates ranging from 8.7% to 21.1% for WEB [15,19,20,41] and 2.3% to 9.3% for FD [17,18].

Within this cohort, six (2.2%) patients experienced a permanent deficit postoperatively. The modified Rankin Score is a frequently used evaluation tool for the clinical outcome after neurosurgical interventions; we therefore performed a subanalysis, which indicated that only two (0.7%) patients should be classified as having an unfavorable outcome (mRS > 2), while the remaining four (1.5%) with a permanent deficit should be classified as having a favorable outcome (mRS 0–2) [22]. Procedure-related complications are also observed in endovascularly treated MCA aneurysms: Iosif et al. [36] reported a 6.9% incidence of procedure-related ischemic events in FD, while De Leacy et al. [20] documented a 6.8% occurrence of permanent deficits in cases involving WEB. In the context of FD for treating MCA aneurysms, thromboembolic events have been reported to occur with a frequency ranging from 8% to ~17% according to findings by Diestro et al. and Salem et al. [17,18]

The mortality rate of 0.7% is attributed to two postoperative deaths unrelated to the clipping procedure. Patient A experienced a fulminant pulmonary embolism, while Patient B developed fatal sepsis, both ultimately resulting in death.

Although Fisher’s exact test did not identify an association between ICG angiography and pNND, it is noteworthy—as highlighted in Table 9—that no cases of pNND were reported when ICG angiography was used in conjunction with conventional angiography. The additional intraoperative benefit of ICG angiography has already been demonstrated several times [42,43].

The logistic regression analysis indicates that patients operated on more recently (year of operation) tend to experience fewer instances of pNND (*p* = 0.009). This temporal trend can likely be attributed to the increasing experience and continuous improvement in both surgical techniques and the ongoing enhancement of technical capabilities in the intraoperative setup. The consistent integration of intraoperative neuromonitoring—high-resolution intraoperative angiography in the hybrid operating room—combined with ICG angiography, is a possible contributor to this advancement. In addition to the continuous progress in endovascular treatment methods, the results demonstrate that microsurgical treatment is also progressively improving in its outcomes in recent years. The incidence of postoperative SSI exhibited a similar trend after stepwise variable selection, with a reduction in cases (*p* = 0.001) over time. Furthermore, the results indicate that older patients are more likely to experience postoperative SSI (*p* = 0.04). This finding suggests that the probability of developing postoperative SSI increases with each additional year of age within this cohort. This is a critical consideration for clinical practice, particularly when evaluating risk factors in the treatment and monitoring of elderly patients. Compared to the multicenter analysis of Drexler et al., the incidence of postoperative SSI with 1.5% (benchmark cutoff: ≤2.7%) is lower than in the benchmark study [44].

With regard to the occurrence of postoperative epileptic seizures, our result of 4.0% is comparable with the available literature (between 2.6% and 15.7%) [45]. Postoperative epileptic seizures are associated with prolonged hospitalization, and the rate of epileptic seizures after endovascular treatment of UIAs is likely to be lower than after microsurgical clipping according to Hoh et al. (6.2% vs. 9.2%), although no distinction was made between the respective endovascular treatment methods [46].

The results of this cohort show that postoperative ICH occurred in 1.1% of the cases as a consequence of the surgical approach employed, necessitating postoperative surgical revision. Importantly, in each of these cases, aneurysm-related secondary bleeding could be ruled out. When comparing our findings to a similar large series published by Metayer et al., the incidences are comparable (1.1% vs. 1.0%), reinforcing the consistency of our results with the existing literature [28].

In summary, our results demonstrate a remarkably high rate of aneurysm occlusion and a notably low rate of retreatment, exceeding the efficacy of endovascular therapy approaches described in the existing literature. This is corroborated by a recent meta-analysis by Tocacelli et al., which compared microsurgical clipping with endovascular techniques for MCA aneurysms, reinforcing our findings [10]. Consequently, these data support the position that microsurgical clipping should be considered the treatment of choice for unruptured MCA bifurcation aneurysms.

## 5. Limitations

The retrospective nature of data collection represents a notable limitation of this study, inherently allowing for potential confounding variables or biases. Although all patients underwent surgery performed by various experienced neurosurgeons, it remains a single-center experience and therefore carries the risk of selection or performance bias. Additionally, the surgeons’ experience over time might have influenced outcomes, although this factor was not systematically assessed. The study exclusively focused on microsurgical interventions, thus omitting any cases from the registry that were treated with endovascular approaches. This decision was made to maintain the study’s focus on providing insights into the current microsurgical management of unruptured MCA bifurcation aneurysms, rather than aiming to demonstrate therapeutic superiority. The representation of large or giant aneurysms in the registry appears to be lower compared to other databases, making it difficult to generalize the findings to more complex MCA bifurcation cases. Further, the evaluation of postoperative neurocognitive outcomes would be of interest, but this information could not be obtained from the retrospective data. Another limitation of this study is the lack of an independent audit or external data collection, which carries a substantial risk of reporting bias.

## 6. Conclusions

The favorable outcomes observed in our study align well with existing benchmarks. This includes a low retreatment rate (0.7%), coupled with a high occlusion rate (99.3%) and a notably low incidence of unfavorable outcomes (0.7%). Continuous enhancements over time, aided by the integration of intraoperative adjuncts (such as intraoperative ICG angiography or intraoperative neuromonitoring), have contributed to a consistent improvement in clinical outcomes. This trend is exemplified by the absence of detectable pNND in the era of ICG angiography. Further prospective investigations, particularly randomized studies comparing the outcomes of microsurgical and endovascular interventions, are warranted.

## Figures and Tables

**Table 1:** Descriptive statistics of preoperative patient- and aneurysm-specific parameters. For categorial variables, absolute frequencies (percent) are reported. There were no missing values. ADPKD = autosomal dominant polycystic kidney disease, SAH = subarachnoidal haemorrhage, max. = maximal, mm = millimeter, prev. = previously.

Preoperative Parameters		Value
Number of aneurysms (n)		272
Patient-specific parameters		
Sex	female	196 (72.06%)
	male	76 (27.94%)
Mean Age in years (standard deviation)		55.08 (10.4)
ADPKD		5 (1.84%)
Unrelated SAH in anamnesis		42 (15.44%)
Aneurysm-specific parameters		
Side	right	151 (55.51%)
	left	121 (44.49%)
Size	Small (<10 mm)	242 (88.97%)
	Large (10–25 mm)	29 (10.66%)
	Giant (>25 mm)	1 (0.37%)
Max. Diameter mean in mm (range)		5.81 (2–25)
Prev. Coiled	No	265 (97.43%)
	Yes	7 (2.57%)
Thrombosed	No	263 (96.69%)
	Yes	9 (3.31%)
Blebs	No	219 (80.51%)
	Yes	53 (19.49%)
Fusiform	No	268 (98.53%)
	Yes	4 (1.47%)

**Table 2:** Intraoperative parameters. For categorical variables, absolute frequencies (percent of non-missing values) are reported. MEP = motor evoked potentials, SSEP = somatosensory evoked potentials, ICG = indocyanine green angiography.

Intraoperative Parameters	Value (%)
Reposition of clips	60 (22.39%)
Average number of clips	1.46
Intraoperative Rupture of aneurysm	8 (2.96%)
Temporary Clipping	38 (14.13%)
Intraoperative Neuromonitoring (MEP, SSEP)	68 (25%)
Intraoperative ICG angiography	102 (37.5%)

**Table 3:** Radiological and Clinical Outcome Parameters. For categorical variables, absolute frequencies (percent of non-missing values) are reported; DSA = digital subtraction angiography, RROC = Raymond–Roy occlusion classification, ICH = intracerebral hematoma, NND = new neurological deficit, MRI = magnetic resonance imaging, SSI = surgical site infection, cSDH = chronic subdural hematoma, SAH = subarachnoidal haemorrhage; a = data missing for four patients.

Radiological Outcome Parameters		Absolut Frequency (%)
Intra-/Postoperative DSA		268 (98.53%)
Postoperative occlusion rate (a)		
RROC Class I		266 (99.25%)
RROC Class II		0
RROC Class III		2 (0.75%)
**Clinical outcome parameters**		**Absolut frequency (%)**
	Postoperative ICH	3 (1.1%)
	New neurological deficit (NND)	19 (6.99%)
	permanent NND	6 (2.21%)
	mRS 0–2 („favorable outcome“)	4 (1.47%)
	mRS 3–5 („unfavorable outcome“)	2 (0.74%)
	transient NND	13 (4.78%)
	Epileptic seizure postoperative	11 (4.04%)
	Surgical site infection (SSI)	4 (1.47%)
	Chronic subdural hematoma (cSDH)	3 (1.1%)
	Pulmonary embolism	2 (0.74%)
	Recurrent Aneurysm (follow-up)	2 (0.74%)
	Retreatment (follow-up)	2 (0.74%)
	SAH in follow-up	1 (0.37%)
	Perioperative Death	2 (0.74%)

**Table 4:** This table reports the results of the logistic regression model for the outcome parameter NND. Stepwise variable selection was performed using the Akaike Information Criterion (AIC). Fusiform is not used as regressor due to separation. NND = new neurological deficit; ADPKD = autosomal dominant polycystic kidney disease, ICG = indocyanine green angiography, No. = number.

	Estimate	Std. Error	z-Value	*p*-Value
**New neurological Deficit (NND)**				
Intercept	−2.546	0.860	−2.961	0.003
Sex (male)	−0.243	0.628	−0.387	0.699
Age (in years)	0.046	0.029	1.596	0.111
ADPKD (yes)	0.753	1.935	0.389	0.697
Side (left)	0.837	0.559	1.499	0.134
Previously Coiled (yes)	0.947	1.195	0.792	0.428
Size—max. Diameter mean in mm	0.064	0.085	0.756	0.450
Thrombosed (yes)	1.399	1.164	1.202	0.230
Blebs (yes)	−0.575	0.756	−0.761	0.447
Intraoperative Neuromonitoring (yes)	0.499	0.912	0.548	0.584
Intraoperative ICG angiography (yes)	0.325	1.030	0.315	0.753
No. of Clips	0.176	0.289	0.610	0.542
Reposition of clips (yes)	0.545	0.642	0.849	0.396
Intraoperative Rupture of aneurysm (yes)	0.122	1.398	0.087	0.930
Temporary Clipping (yes)	0.121	0.747	0.161	0.872
Year of Surgery	−0.149	0.098	−1.527	0.127
	***Logistic regression model after stepwise variable selection:***			
Intercept	−3.388	0.432	−7.836	<0.001
Side (left)	0.897	0.531	1.689	0.091
Size—max. Diameter mean in mm	0.168	0.052	3.246	0.001

**Table 5:** This table reports the results of the logistic regression model for the outcome parameter pNND. Previously coiled, fusiform and intraoperative ICG angiography were not used as regressors due to separation. Stepwise variable selection was performed using the Akaike Information Criterion (AIC). NND = new neurological deficit; pNND = permanent new neurological deficit, ADPKD = autosomal dominant polycystic kidney disease, No. = number.

	Estimate	Std. Error	z-Value	*p*-Value
**Permanent NND (pNND)**				
Intercept	−1.618	1.701	−0.951	0.342
Sex (male)	−2.033	1.722	−1.181	0.238
Age (in years)	0.047	0.066	0.719	0.472
ADPKD (yes)	3.212	4.541	0.707	0.479
Side (left)	0.500	1.033	0.484	0.628
Size—max. Diameter mean in mm	−0.098	0.200	−0.490	0.624
Thrombosed (yes)	2.157	1.944	1.110	0.267
Blebs (yes)	0.424	1.277	0.332	0.740
Intraoperative Neuromonitoring (yes)	3.068	1.834	1.673	0.094
No. of Clips	−0.119	0.740	−0.160	0.873
Reposition of clips (yes)	1.547	1.238	1.250	0.211
Intraoperative Rupture of aneurysm (yes)	2.517	2.341	1.075	0.282
Temporary Clipping (yes)	−2.920	2.393	−1.220	0.222
Year of Surgery	−0.461	0.205	−2.249	0.025
	***Logistic regression model after stepwise variable selection:***			
Intercept	−1.255	1.012	−1.240	0.215
Sex (male)	−1.999	1.399	−1.429	0.153
Intraoperative Neuromonitoring (yes)	3.093	1.356	2.281	0.023
Intraoperative Rupture of aneurysm (yes)	3.196	1.622	1.971	0.049
Temporary Clipping (yes)	−2.448	1.892	−1.294	0.196
Year of Surgery	−0.403	0.154	−2.615	0.009

**Table 6:** This table reports the results of the logistic regression model for the outcome parameter tNND. ADPKD, fusiform, and intraoperative rupture of the aneurysm were not used as regressors due to separation. Stepwise variable selection was performed using the Akaike Information Criterion (AIC). NND = new neurological deficit; tNND = transient new neurological deficit, ADPKD = autosomal dominant polycystic kidney disease, ICG = indocyanine green angiography, No. = Number.

	Estimate	Std. Error	z-Value	*p*-Value
**Transient NND (tNND)**				
Intercept	−3.507	1.048	−3.346	0.001
Sex (male)	0.227	0.709	0.320	0.749
Age (in years)	0.064	0.036	1.790	0.073
Side (left)	0.828	0.667	1.241	0.215
Previously Coiled (yes)	1.428	1.214	1.177	0.239
Size—max. Diameter mean in mm	0.079	0.104	0.763	0.446
Thrombosed (yes)	1.432	1.458	0.982	0.326
Blebs (yes)	−1.207	1.160	−1.040	0.298
Intraoperative Neuromonitoring (yes)	−0.964	1.072	−0.899	0.368
Intraoperative ICG angiography (yes)	1.675	1.155	1.449	0.147
No. of Clips	0.315	0.364	0.865	0.387
Reposition of clips (yes)	0.022	0.857	0.025	0.980
Temporary Clipping (yes)	0.527	0.871	0.605	0.545
Year of Surgery	−0.147	0.123	−1.194	0.232
	***Logistic regression model after stepwise variable selection:***			
Intercept	−3.305	0.343	−9.637	<0.001
Size—max. Diameter mean in mm	0.149	0.057	2.600	0.009

**Table 7:** This table reports the results of the logistic regression model for the outcome parameter epileptic seizures postoperative. Previously coiled and thrombosed were not used as regressors due to separation. Stepwise variable selection was performed using the Akaike Information Criterion (AIC). ADPKD = autosomal dominant polycystic kidney disease, ICG = indocyanine green angiography, No. = number.

	Estimate	Std. Error	z-Value	*p*-Value
**Epileptic seizures postoperative**				
Intercept	−2.457	1.098	−2.237	0.025
Sex (male)	1.994	0.763	2.614	0.009
Age (in years)	0.019	0.038	0.498	0.619
ADPKD (yes)	4.381	2.026	2.162	0.031
Side (left)	−0.667	0.795	−0.839	0.402
Size—max. Diameter mean in mm	0.098	0.128	0.770	0.441
Fusiform (yes)	2.610	1.683	1.551	0.121
Blebs (yes)	−0.532	0.943	−0.564	0.573
Intraoperative Neuromonitoring (yes)	0.749	1.149	0.652	0.515
Intraoperative ICG angiography (yes)	3.130	1.618	1.934	0.053
No. of Clips	−0.658	0.604	−1.090	0.276
Reposition of clips (yes)	0.552	0.866	0.638	0.523
Intraoperative Rupture of aneurysm (yes)	−2.704	2.507	−1.079	0.281
Temporary Clipping (yes)	1.674	0.877	1.909	0.056
Year of Surgery	−0.312	0.149	−2.092	0.036
	***Logistic regression model after stepwise variable selection:***			
Intercept	−3.447	0.872	−3.953	<0.001
Sex (male)	1.736	0.729	2.381	0.017
ADPKD (yes)	3.227	1.555	2.076	0.038
Fusiform (yes)	3.358	1.484	2.263	0.024
Intraoperative ICG angiography (yes)	2.779	1.439	1.932	0.053
Temporary Clipping (yes)	1.560	0.747	2.089	0.037
Year of Surgery	−0.258	0.121	−2.125	0.034

**Table 8:** This table reports the results of the logistic regression model for the outcome parameter SSI. Stepwise variable selection was performed using the Akaike Information Criterion (AIC). ADPKD, previously coiled, thrombosed, fusiform, temporary clipping, and intraoperative rupture of the aneurysm were not used as regressors due to separation. SSI = surgical site infection, ADPKD = autosomal dominant polycystic kidney disease, ICG = indocyanine green angiography, No. = number.

	Estimate	Std. Error	z-Value	*p*-Value
**Surgical Site Infection (SSI)**				
Intercept	−1.153	1.204	−0.957	0.338
Sex (male)	0.804	0.885	0.908	0.364
Age (in years)	0.119	0.052	2.267	0.023
Side (left)	−0.871	0.896	−0.972	0.331
Size—max. Diameter mean in mm	−0.165	0.144	−1.144	0.253
Blebs (yes)	0.360	0.930	0.387	0.699
Intraoperative Neuromonitoring (yes)	3.127	1.267	2.468	0.014
Intraoperative ICG angiography (yes)	5.600	2.269	2.467	0.014
No. of Clips	0.174	0.680	0.256	0.798
Reposition of clips (yes)	0.753	1.078	0.698	0.485
Year of Surgery	−0.776	0.245	−3.173	0.002
	***Logistic regression model after stepwise variable selection:***			
Intercept	−1.046	0.682	−1.534	0.125
Age (in years)	0.091	0.044	2.069	0.039
Intraoperative Neuromonitoring (yes)	2.309	1.013	2.280	0.023
Intraoperative ICG angiography (yes)	4.914	1.969	2.496	0.013
Year of Surgery	−0.662	0.200	−3.305	0.001

**Table 9:** Subanalysis of the effect of ICG on permanent neurological deficits (pNND). Crosstable of absolute frequencies and *p*-value of Fisher’s exact test (right column). ICG = indocyanine green angiography, pNND = permanent new neurological deficit.

		pNND		*p*-Value
		Yes	No	
**ICG Angiography**	yes	0	102	0.087
	no	6	163	

## Data Availability

The data that support the findings of this study are available on reasonable request from the corresponding author Philip Rauch. The data are not publicly available due to privacy and ethical restrictions.

## Abbreviations

cSDH	chronic subdural hematoma
DSA	Digital Subtraction Angiography
FD	Flow-Diverter
ICG	Indocyanine green angiography
ICH	intracerebral hematoma
MCA	middle cerebral artery
MEP	motor evoked potential
MRI	Magnetic Resonance Imaging
NND	new neurological deficit
pNND	permanent new neurological deficit
RROC	Raymond–Roy occlusion classification
SAH	subarachnoid hemorrhage
SD	standard deviation (SD)
SSEP	somatosensory evoked potential
SSI	surgical site infection
tNND	transient new neurological deficit
UIA	unruptured intracranial aneurysm
WEB	Woven Endo Bridge
